# Response to Reich and Lipner, “Defining an onychomycosis cohort: The importance of mycologic sampling”

**DOI:** 10.1016/j.jdin.2026.03.005

**Published:** 2026-03-20

**Authors:** Sonya V. Gupta, Kevin Chen, Jasmine Rana

**Affiliations:** a Stanford University School of Medicine, Palo Alto, California; b Department of Dermatology, Stanford Medicine, Redwood City, California

**Keywords:** antifungal, ciclopirox, efinaconazole, fluconazole, itraconazole, nail disease, nail fungal infection, onychomycosis, tavaborole, telemedicine, terbinafine

*To the Editor:* We thank Reich and Lipner^1^ for their comments, and we agree with the study limitations they raise. Our study attempted to characterize real-world antifungal-prescribing behaviors for onychomycosis between video and in-person visits.^2^ Our analysis may not capture variable clinical workflows across multiple specialties. In fact, an antecedent onychodystrophy code at the time of nail clipping, for example, may precede a first-time coded onychomycosis diagnosis, complicating analysis further. We do clarify that our 3-month comparison was intended to capture if confirmatory testing (typically does not take >3 weeks to result) might influence treatment strategy not to assess treatment response.

Recent DataDerm data remind us that confirmatory testing remains underused in practice, with less than a quarter of patients receiving such testing.^3^ We wonder if this could be further exacerbated by virtual care, and this deserves further study. We hope that our findings inspire ongoing research, particularly as polymerase chain reaction–based diagnostics become more widely available and antifungal resistance continues to emerge as a pressing concern.

## Conflicts of interest

None disclosed.
